# Medical Extracts

**Published:** 1884-03

**Authors:** 


### Medical Extracts.

---

### A New Treatment of Cancer.

Not long ago Professor Nussbaum treated a case of cancer of the mamma, with so strong a tendency to hemorrhage that each dressing of the ulcer threatened the woman's life. As the patient was nearly moribund from the frequent loss of blood, he surrounded the tumour with a strong subcutaneous ligature, which he drew together with all his strength and then tied. The result was not only an arrest of the hemorrhage and a speedy rally of the patient, but a constant decrease of the tumour to one-quarter of its size, when the ulcer began to cicatrize. He now proposes to prevent the feeding of these tumors by their peripheral nutrient vessels in the manner indicated. The blood which supplies the tumor through its base, while it does not absolutely starve it, is, however, sufficient to keep it stationary. In order to accomplish the desired interruption of the blood supply from the periphery, he urges the formation of a furrow around the tumor down to the fascia, about one centimetre wide, made by means of a thermo-cautery.—*The Therapeutic Gazette*, Dec., 1883.

### The late Dr. J. Marion Sims.

At the last meeting of the Newport (R.I.) Medical Society Dr. Samuel W. Francis, Vice-President, presented the following resolutions, which were adopted :—

*Whereas*, It has pleased Almighty God to remove from this earth Dr. J. Marion Sims; therefore

*Resolved*, That, in his death, Science has lost a star; Surgery a pioneer; Gynecology an originator; America one of her philanthropists; and the fair sex their best friend.

*Resolved*, That the name of J. Marion Sims will live till woman dies.

*Resolved*, That the boldness of his operations was only equalled by his tenderness and sympathy at the bedside.

*Resolved*, That, as he belonged to the world, both hemispheres will mourn his loss and cherish his memory.

*Resolved*, That a copy of these resolutions be transmitted to the family.

### The Beef Tea Fallacy.

From time immemorial beef tea has been one of the standard beverages and nutriments of the sick room, and were its history fully written, we have little doubt that it would show a great mortality attending its use. By filling the stomach to distension it is capable of allaying hunger, while the patient may be literally starving, and doubtless many a fatal case of disease of an adynamic type is ascribable to the fallacious food with which it has been sought to support the vital powers, rather than to the disease itself.

Look at the meat after the beef tea has been prepared from it. The bulk of the solids of the meat is myosin, which is the substance we intend to pay for when we buy butcher's meat; the substance we mean to feed upon when we eat the beef steak. After the preparation of beef tea this myosin is thrown away, a little the worse for its treatment, a little tough, insipid, deprived of its salts, yet containing all the strength of the meat. The tea has removed from it no nutritious substance, excepting only the mineral salts. Four fluid ounces of beef tea were dried in a water bath, not quite perfectly, in order to avoid any possible destruction and hence loss, but rather to allow the error to tell in favour of the beef tea. The residue weighed 51 grains, that is, four fluid ounces of beef tea contained 51 grains of solid matter.

Professor Baumgarten's experiments are the most convincing with which we have been favoured for some time, of the fact of the almost absolute uselessness of beef tea and the various extracts and essences of beef which are largely sold, and which the people, and too large a proportion of the profession, are made to believe to be foods or substances capable of sustaining life and repairing waste of tissue. Beef tea and the various extracts and essences referred to, are useful only in so far as they stimulate appetite and facilitate the digestion of such food as may be given in connection with them. This is their only legitimate function, and when they are depended upon to the exclusion of true food, the result is only disaster to the confiding patient.—*Loc. Cit.*, Oct., 1883.

### Antiseptic Sugar Dressings for Wounds.

Dr. Fischer reports in the *Centralblatt für Chiurgie* for August 25, 1883, that he and Professor Lücke have used this application in several instances with satisfactory results. They have used a mixture containing equal parts of sugar and napthaline, and one of iodoform one part to sugar five parts. The mixture is rubbed into gauze, which is applied over the line of suture, or, where there is a loss of integument, the sugar is strewn directly upon the tissues. Sugar cushions were prepared and used in the same way as peat or sublimate sand cushions. The dressings may be left *in situ* for eight or ten days. The secretions are diffused equally throughout the mass of sugar, except when the layer is too thick it sometimes becomes lumpy. There was never any bad odor about the dressings, even when they had been on a long time. Granulation proceeded finely under the sugar; there was no bleeding, and cicatrization was quickly accomplished.

### Extirpation of the Lung.

Dr. Domenico Biond has removed one entire lung fifty-seven different times in animals (sheep, dogs, cats, etc.), of which number thirty survived. In five instances, in which only portions of one or both lungs were extirpated, the animals all recovered. The failure, in the thirty cases of death, the experimenter attributed in great part to the neglect of proper antiseptic precautions.—*L'Union Médicale*, September 2, 1883.

### On the Intra-Venous Injection of Saline Solutions as a substitute for transfusion of Blood.

The use of saline injections in Asiatic cholera in the early part of this century demonstrated the safety of such a procedure, and likewise its inefficiency in checking the career of that disease. Within a few years, however, this method has risen to the level of a life-saving measure, as a substitute for the transfusion of blood in conditions of acute anæmia and collapse. Of nineteen patients who have been subjected to the operation, when at the point of death, thirteen have entirely recovered; in three death was averted, but occurred later; and in the remainder only a temporary improvement was effected. [A table of the cases is given.]

Dr. Jennings recommends this solution: chloride of sodium, 50 grs.; chloride of potassium, 3 grs.; sulphate of soda, 2.5 grs.; carbonate of soda, 2.5 grs.; phosphate of soda ($Na_3Po_4$) 2 grs.; dissolved in twenty ounces of water at 100° F., with the addition of 2 drachms of absolute alcohol, to be injected by means of a siphon of rubber tubing or a brass syringe.

The salts are kept in a small phial. A trocar and also a blunt stylet are added, in order that one may puncture the vein with the former, or after opening it guide the canula into it on the latter instrument. An ordinary glass or metallic irrigator, or a funnel with tubing attached will do about as well. Let the solution (water, ℥ xxxij.; chloride of sodium, ʒ jss.; carbonate of soda, gr. xv.), warmed to 100° F., flow from a height of two or three feet in the course of fifteen or twenty minutes. The canula should be no larger than a medium-sized aspirator needle, one-sixteenth inch in diameter; the apparatus disinfected with three per cent. solution of carbolic acid and antiseptic precautions observed in operating. Choose the arm in which a well-distended vein can be seen at the bend of the elbow, and failing to find one secure the radial artery, and inject the fluid into its central end.—*N. Y. Med. Record*, January 5th, 1884.

